# Hepatitis B virus inhibits intrinsic RIG-I and RIG-G immune signaling via inducing miR146a

**DOI:** 10.1038/srep26150

**Published:** 2016-05-23

**Authors:** Zhaohua Hou, Jian Zhang, Qiuju Han, Chenhe Su, Jing Qu, Dongqing Xu, Cai Zhang, Zhigang Tian

**Affiliations:** 1 Institute of Immunopharmaceutical Sciences, School of Pharmaceutical Sciences, Shandong University, China; 2 Collaborative Innovation Center for Diagnosis and Treatment of Infectious Diseases, State Key Laboratory for Diagnosis and Treatment of Infectious Diseases, First Affiliated Hospital, College of Medicine, Zhejiang University, Hangzhou, Zhejiang 310003, China; 3 Institute of Immunology and The CAS Key Laboratory of Innate Immunity and Chronic Disease, School of Life Sciences and Medical Center, University of Science and Technology of China, Hefei, Anhui 230027, China

## Abstract

Previous studies showed that hepatitis B virus (HBV), as a latency invader, attenuated host anti-viral immune responses. miRNAs were shown to be involved in HBV infection and HBV-related diseases, however, the precise role of miRNAs in HBV-mediated immunosuppression remains unclear. Here, we observed that down-regulated RIG-I like receptors might be one critical mechanism of HBV-induced suppression of type I IFN transcription in both HBV^+^ hepatoma cell lines and liver cancer tissues. Then, miR146a was demonstrated to negatively regulate the expression of RIG-I-like receptors by directly targeting both RIG-I and RIG-G. Further investigation showed that antagonizing miR146a by anti-sense inhibitors or sponge approach accelerated HBV clearance and reduced HBV load both *in vitro* and in a HBV-carrying mouse model. Therefore, our findings indicated that HBV-induced miR146a attenuates cell-intrinsic anti-viral innate immunity through targeting RIG-I and RIG-G, and silencing miR146a might be an effective target to reverse HBV-induced immune suppression.

It is well-known that both host and viral factors determine the outcome of HBV infection in the host^1^, whereas impaired immune response is mostly responsible for HBV chronicity, latency, reactivity and deterioration. Innate immune system recognizes virus components by pattern recognition receptors (PRRs) and acts as the first line of defense to limit viral replication in host cells. Many PRR members are indispensible for anti-HBV immune responses^2^, which promotes type I IFN production, and then avoids acute viral expansion or long-term chronic infection^3^,^4^. However, in the case of HBV, it can interfere with multifaceted mechanisms to evade TLR/RLR-mediated antiviral signaling pathways^5^, among which counteracting type I IFN pathway is an essential one.

In previous study, we found that HepG2.2.15 cells produced less IFN-β upon poly(I:C) stimulation compared with the parent HepG2 cells^6^. HBV polymerase over-expression could weaken RIG-I- and TLR3-induced IFN-β secretion in HepG2 cells^7^. Moreover, HBx may block RIG-I signalling by different ways, including disturbing the interaction between IPS-1 and RIG-I^8^,^9^, and the interaction between RIG-I and TRIF as a deubiquitinating enzyme^10^. In view of these evidences, many achievements have been made to explore the molecular mechanisms of HBV immune evasion and create approaches for controlling HBV infection, but whether epigenetic regulation such as posttranscriptional modification is involved in this process remains largely unknown.

MicroRNAs (miRNAs), a big family of small single strand RNAs (~18 to 24 nucleotides in length), play important roles in regulating gene expression at posttranscriptional level. Until now, many miRNAs have been identified to regulate HBV life cycle or influence the outcome of HBV infection^11^,^12^, including a well-recognized immuno-miR, miR146a^13^. MiR146a controls lymphocyte development, and were also involved in anti-viral and anti-tumor innate immune responses^14^. Although some independent studies have described miR146a was up-regulated in HBV positive HCC cells by miRNA profile analysis^12^,^15^,^16^,^17^, the details about how miR146a was involved in the progression of HBV infection was rarely mentioned. In 2013, Meng’s group reported that miR146a feedback suppressed cytokine production and cytotoxicity by targeting STAT1 in CD4^+^ and CD8^+^ T cells from CHB patients^18^, suggesting that miR146a attenuates adaptive anti-HBV immunity by down-regulating target genes in lymphocytes. At the same time, we investigated the role of miR146a in HBV-associated interferon resistance in hepatocytes^19^. But whether miR146a can regulate anti-HBV innate immune response in hepatocytes, the host cell of HBV, is largely unknown.

To understand the precise mechanisms of miR146a in HBV-induced immune suppression, in this study, we found that HBV-induced miR146a could post-transcriptionally inhibit expression of both RIG-I and RIG-I enhancer (RIG-G), leading to suppressing type I IFN production and resulting in impairment of anti-HBV innate immunity. Accordingly, antagonizing miR146a reversed immune tolerance and generated efficient anti-HBV immunity.

## Results

### HBV infection inhibited the expression of RIG-I like receptors

**Figure 1:**
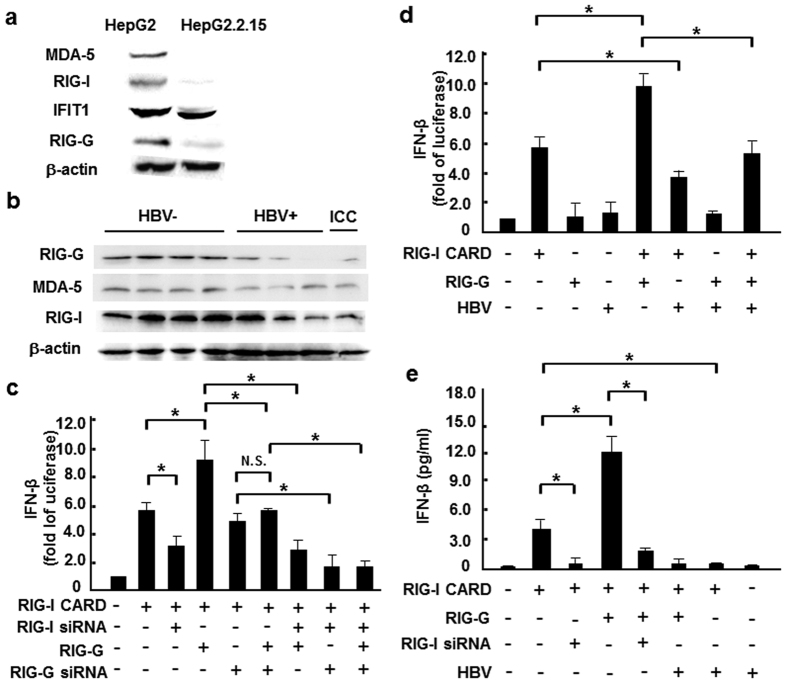
HBV infection inhibited expression of RIG-I like receptors. (**a**) Western blot analysis of RIG-I, MDA-5, IFIT1 and RIG-G expression levels in HepG2 and HepG2.2.15 cells. (**b**) Western blot assay of RIG-I, MDA-5 and RIG-G protein levels in liver paracancerous tissues from 4 HBV^+^, 3 HBV^−^ HCC and 1 intrahepatic cholangiocarcinoma (ICC) patients. (**c**) HepG2 cells were co-transfected with 0.05 μg RIG-I CARD, IFN-β–luc and pRL-TK reporter vectors, as well as RIG-G constructs or 100 nM RIG-I/RIG-G siRNA. After 36 hours, luciferase activity was measured. (**d**) RIG-I CARD/RIG-G constructs (0.5 μg/ml) and pAAV/HBV1.2 plasmid were co-transfected into HepG2 cells, together with the IFN-β–luc and pRL-TK reporter vectors, and luciferase activity was measured 36 hours later. (**e**) RIG-I CARD/RIG-G constructs (0.5 μg/ml) and pAAV/HBV1.2 plasmid (0.5 μg/ml), as well as 100 nM RIG-I siRNA were co-transfected into HepG2 cells, together with the IFN-β–luc and pRL-TK reporter vectors, and ELISA method was used to measure IFN-β production 36 hours later. Data are expressed as the mean ± SD from at least 3 independent experiments. *p < 0.05: versus the control vector–transfected group.

To identify whether PRRs in liver parenchymal cells were influenced by HBV infection, firstly the expression of RNA-sensing receptors in HBV^+^ and HBV^−^ hepatocytes were compared, including RIG-I, MDA5 and TLR3/7, as well as the new viral RNA receptor IFIT1^20^ and the enhancer of the RIG-I signaling pathway RIG-G^21^. Similar to previous reports^22^,^23^, we found that cytoplasmic RNA receptors were down-regulated in HBV^+^ HepG2.2.15 cells (Fig. 1a) compared to HepG2 cells, but no significant changes were observed in TLR expression (data not shown). Consistently, RIG-I, RIG-G and MDA-5 protein levels in HBV^+^ human liver paracancerous tissues were also lower than in HBV^−^ tissues (Fig. 1b). Then, we tried to explore whether the depressed RIG-I pathway would lead to lower type I IFN production. As expected, RIG-G over-expression increased RIG-I CARD-induced IFN-β transcription at approximately 2-folds, which would be blocked by silencing RIG-I (Fig. 1c), indicating RIG-G was a downstream enhancer of the RIG-I signaling pathway in hepatocytes. Furthermore, transfection of the vector containing HBV genome counteracted the synergy between RIG-G and RIG-I, and restrained RIG-I–mediated IFN-β transcriptional activity (Fig. 1d,e). These findings suggested that HBV infection directly suppressed RLR pathway in hepatocytes, which could interrupt the downstream type I IFN production.

### miR146a level correlated with HBV infection both *in vitro* and *in vivo*

**Figure 2:**
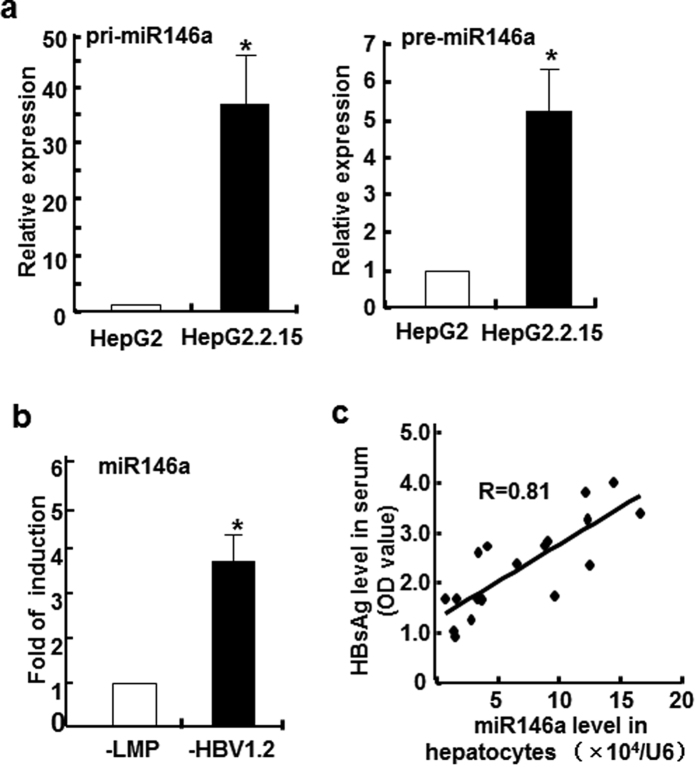
miR146a level correlated with HBV infection both *in vitro* and *in vivo*. (**a**) The primary transcript (left) and precursor (right) of miR146a in HepG2 and HepG2.2.15 cells were assessed by qRT-PCR. (**b**) HBV genome-transfected HepG2 cells were established by transfecting HepG2 cells with the LMP-HBV1.2 plasmid, followed by puromycin selection. RNA from these transfected and control cells was isolated, and miR146a expression levels were evaluated by qRT-PCR. miR146a levels in HBV^+^ cells were expressed as the fold of the level in control cells. GAPDH was used as the internal control. (**c**) HBV-carrying BALB/c mice were established by hydrodynamic injection of pAAV/HBV1.2 at a dose of 6 μg per mouse. After 2 weeks, serum samples were harvested, and HBsAg levels were measured by ELISA. Simultaneously, miR146a levels in primary hepatocytes isolated by collagenase perfusion were measured as above. Data are expressed as the mean ± SD from at least 3 independent experiments. *p < 0.05: versus HepG2-vector cells.

In previous study, we found that the expression of two miR146 family members, miR146a and miR146b, increased more than 200-folds in HepG2.2.15 cells than HepG2 cells, and similar phenomenon was also observed on human HBV^+^ liver samples compared to HBV negative ones^19^. Here, we further confirmed this key point by another *in vitro* method, ribonuclease protection assay (RPA). As shown in Fig. S3, the levels of mature miR146a in HepG2.2.15 cells were relatively high, up to those in miR146a positive leukemia cells (NKL cells), whereas miR146a levels in HepG2 cells were very low. Meanwhile, compared to HepG2 cells, miR146a primary transcript (~200 bp) and its stem-loop precursor in HepG2.2.15 cells were increased approximately 25- and 5-fold, respectively (Fig. 2a). Furthermore, miR146a expression could be increased nearly 4-folds in HepG2 cells by HBV genome (HepG2-LMP-HBV1.2) transfection (Fig. 2b). To confirm the role of HBV on miR146a expression *in vivo*, a HBV-bearing mice model was established by hydrodynamic tail-vein injection of pAAV/HBV1.2 plasmid. As showed in Fig. 2c, a positive correlation between serum HBsAg levels and miR146a levels in primary hepatocytes was observed. These results further demonstrated that HBV infection would up-regulat miR146a in hepatocytes.

### miR146a suppressed RIG-I like receptor-mediated innate immune response

**Figure 3:**
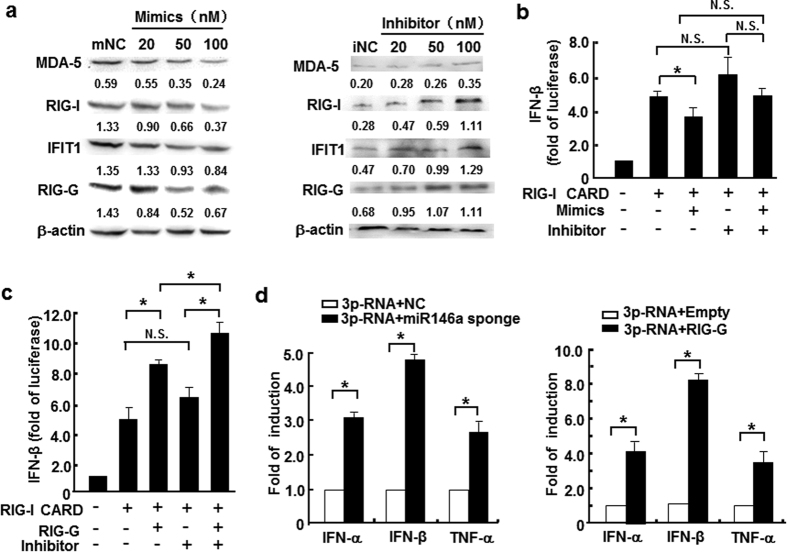
miR146a suppressed RIG-I like receptor- mediated innate immune response. (**a**) RIG-I, MDA-5, IFIT1 and RIG-G protein levels in HepG2 cells treated with miR146a mimics (left), and HepG2.2.15 cells treated with miR146a inhibitors (right) for 48 hours were analyzed by Western blot. One representative of at least 3 independent experiments. (**b**) HepG2 cells were transfected with 0.5 μg/ml RIG-I CARD construct and 100 nM miR146a mimics or inhibitors, together with the IFN-β–luc and pRL-TK reporter vector, and then luciferase activity was measured 36 hours later. (**c**) HepG2.2.15 cells were co-transfected with 0.5 μg/ml of the RIG-I CARD constructs, IFN-β–luc and pRL-TK reporter vectors, as well as RIG-G constructs or 100 nM of miR146a inhibitors, and then luciferase activity was measured 36 hours later. (**d**) HepG2.2.15 cells were transfected with pSIREN-PGK-miR146a-sponge plasmid (left) or pIRESpuro3-RIG-G plasmid (right) at a final dose of 1.5 μg/mL. After 24 hours, these cells were transfected with 3p-RNA at a final concentration of 1 μg/mL. After another 6 hours, the mRNA levels of IFN-α, IFN-β and TNF-α were measured by qRT-PCR. Data are expressed as the mean ± SD from at least 3 independent experiments. Empty, control vector pIRESpuro3; “-” represents miR146a mimic or inhibitor negative control. *p < 0.05: versus negative control RNA or control-vector–transfected group.

To identify whether HBV-induced miR146a influenced PRR-triggered innate immune response, miR146a mimics were introduced into HepG2 cells, and then RIG-I, MDA5, IFIT1 and RIG-G protein levels were analyzed. Interestingly, miR146a mimics decreased the levels of these RNA-sensing receptors in a dose-dependent manner in HepG2 cells (Fig. 3a left), but neither TLR3 nor TLR7 was significantly impacted (data not shown). On the other hand, in HepG2.2.15 cells, miR146a inhibitor enhanced the expression of these RNA-sensing receptors (Fig. 3a right). Meanwhile, an IFN-β biological assay showed that miR146a over-expression suppressed IFN-β transcriptional activation (Fig. 3b); but, miR146a inhibitor enhanced IFN-β induction, which was similar to RIG-G over-expression (Fig. 3c). Furthermore, RIG-I ligand nonsense 5′-triphosphate-RNA (3p-RNA) could trigger more type I IFN and TNF-α expression in HepG2.2.15 cells pre-transfected with miR146a-sponge plasmid (Fig. 3d left) and in HepG2.2.15 cells over-expressing RIG-G (Fig. 3d right), respectively. These evidences suggested that miR146a functioned as an inhibitory regulator for RIG-I pathway, therefore we hypothesized that miR146 might directly interact with RNA-sensing signaling components in hepatocytes.

### miR146a might directly target RIG-I and RIG-G

**Figure 4:**
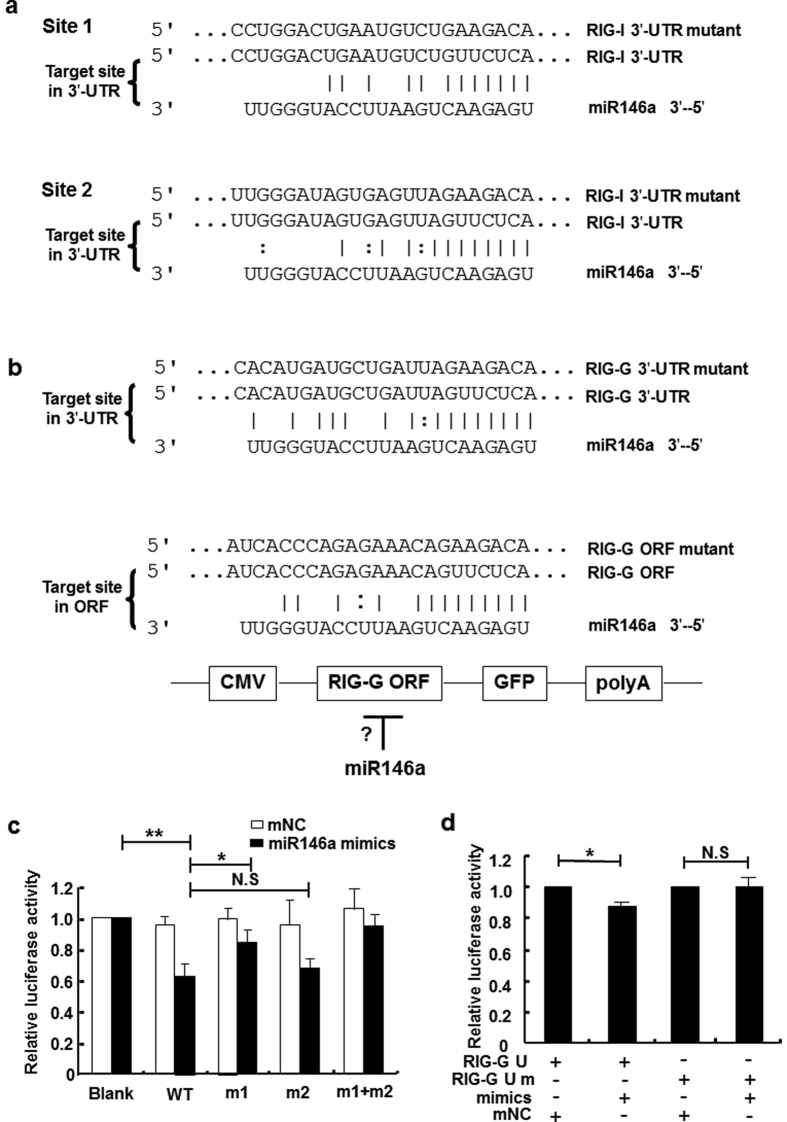
miR146a might directly target RIG-I and RIG-G. (**a**) Sequence alignment between miR146a and the 3′-UTRs of RIG-I. The 3′-UTR of human RIG-I contained 2 potential miR146a binding sites. (**b**) Sequence alignment between miR146a and the 3′-UTRs of RIG-G and ORF region of RIG-G. (**c**) HEK293 cells were transfected with pmiR-reporter-RIG-I 3′-UTR or mutation constructs and miR146a mimics, (m1, contains a mutation in potential binding site 1; m2, contains a mutation in potential binding site 2; m1+m2, contains mutations in both potential binding sites 1 and 2). (**d**) HEK293 cells were co-transfected with pmiR-reporter-RIG-G 3′-UTR or mutation constructs and miR146a mimics or negative control (mNC). Luciferase activity was measured after 36 hours. Data are representative of 3 independent experiments and are expressed as the mean ± SD. *p < 0.05, **p < 0.01.

To test whether miR146a directly targeted the components of RIG-I signal pathway, a set of anti-HBV molecules, including receptors, host restriction factors and ISGs, were selected and submitted to bioinformatic analysis using 4 software programs—PicTar^24^, TargetScan^25^, RNAhybrid^26^ and miRanda^27^. Besides validated targets in the type I IFN signaling pathway, several other genes were predicted to be novel candidates of miR146a targeting (Table 1). Among them, RIG-I possessed two potential miR146a binding sites within its 3′-UTR (Fig. 4a upper). For RIG-G, a 3′-UTR site which had previously been predicted to be a miR146a target but not been validated^28^ (Fig. 4a lower), and another potential miR146a binding site in the ORF (open reading frame) region were predicted at the same time (Fig. 4b). To further identify whether RIG-I and RIG-G were targets of miR146a, we constructed wild-type (WT) RIG-I and RIG-G 3′-UTR regions containing luciferase reporter vectors as well as vectors containing mutant nucleotides corresponding to the putative binding sites (4 bp within the seed region). For RIG-I, we observed that miR146a mimics inhibited luciferase activity from WT RIG-I, but less from the site-1 RIG-I mutant, indicating that site 1 within the RIG-I 3′-UTR was the main binding site for miR146a and site 2 was secondary (Fig. 4c). For RIG-G, miR146a mimics decreased the luciferase activity from WT RIG-G 3′-UTR, but not from the RIG-G mutant (Fig. 4d). In contrast, the potential site within the RIG-G ORF region was not targeted by miR146a (data not shown). These results demonstrated that miR146a could directly target human RIG-I and RIG-G 3′-UTR in hepatocytes, weakening RIG-I–triggered IFN-β transcription.

### Silencing miR146a reversed HBV-induced immune suppression

**Figure 5:**
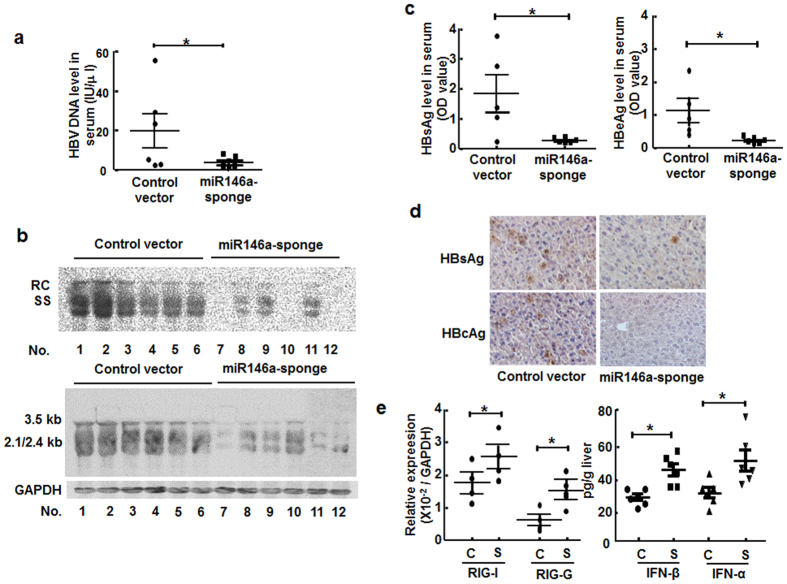
Silencing miR146a reversed HBV-induced immune suppression *in vivo*. HBV-carrying BALB/c mice were injected with pSIREN-PGK-miR146a-sponge plasmid or control vector by hydrodynamic tail-vein injection. Four weeks later, HBV clearance was valuated. (**a**) Serum HBV DNA levels were measured by qRT-PCR. (**b**) Total HBV DNA and RNA levels in mouse liver tissues were analyzed by Southern blotting (upper) and Northern blotting (lower), respectively, with the same HBV probes. Mouse GAPDH was used as the loading control for Northern blotting. (**c**) Serum HBsAg (left) and HBeAg levels (right) were measured by ELISA. (**d**) HBsAg and HBcAg expression in liver tissue were measured by immunohistochemical staining. (**e**) mRNA levels of RIG-I and RIG-G in primary hepatocytes were evaluated by qRT-PCR (left). The concentrations of IFN-α and IFN-β in liver homogenates were evaluated by ELISA (right). C, control vector; S, pSIREN-PGK-miR146a-sponge plasmid. Data represent 2 independent experiments with 6 mice per group. *p < 0.05: versus control-vector–injected group.

Since miR146a inhibited type I IFN-mediated anti-HBV response by targeting STAT1, RIG-I and RIG-G in host cells, we determined whether silencing miR146a would ameliorate the immune response and limit HBV replication *in vitro* and *in vivo*. After transfected with miR146a inhibitor, HBV DNA load in culture supernatant, HBV DNA and RNA amount in HepG2.2.15 cells were decreased significantly (Fig. S2). Subsequently, we employed miR146-sponge plasmid to interfere with endogenous miR146a of hepatocytes in HBV-carrying BALB/c mice. We found serum HBV DNA (Fig. 5a), HBV nucleic acid amount in liver tissue (Fig. 5b), were significantly decreased after miR146-sponge plasmid injection compared to control vector in HBV-carrying mice, accompanied with the down-regulation of HBsAg and HBeAg levels in serum (Fig. 5c), as well as in liver tissues (Fig. 5d). Importantly, RIG-I and RIG-G expression were upregulated by miR146-sponge plasmid treatment (Fig. 5e), accompanied by the elevation of IFN-α and IFN-β production (Fig. 5e). These data indicated that depressing miR146a expression could accelerate HBV clearance and reverse HBV-induced immune suppression *in vivo*.

## Discussion

Several transcriptomic profile analyses showed the limited or even no anti-viral genes were transcribed in the early period after HBV invasion in acute HBV-infected chimpanzee^29^ and CHB woodchuck models^30^, suggesting that HBV can avoid to be recognized or captured by host immune system. Since innate immunity against HBV would influence the prognosis and outcomes of HBV infection^2^, clarifying the underlying molecular mechanisms of anti-HBV innate immune response should be an important topic in HBV immunopathology, and also be benefit to novel anti-HBV drug development.

Although the miRBase database has not yet formally accepted any HBV-encoded miRNA-like small RNA as a member, many miRNAs originated from host cells conceivably regulate HBV gene transcription and replication through directly targeting the virus or by targeting host factors that promote or inhibit HBV life cycle and latency^11^. Besides these HBV-targeting miRNAs, some other host miRNAs were also involved in the pathogenesis of virus-related liver diseases, including acute and chronic hepatitis, liver fibrosis and hepatocellular carcinoma^31^,^32^,^33^. Based on these previous studies, miRNAs were considered to be involved in HBV-host interaction. Several studies have exhibited the relationship between miR-146a and the risk of hepatocellular carcinoma, and demonstrated highly expressed miR-146a decreased the sensitivity of hepatoma cells to IFN-a treatment through targeting SMAD4, resulting in the suppression of apoptosis^34^. Noticeably, miR146a in PBMCs could down-regulate the production of TNF-α, which prevented excessive innate immune activation and subsequent liver damage^35^. Under this circumstance, miR146a may provide a protective effect for liver via avoiding immune attack at acute phase, and reduces the susceptibility to ACLF-HBV. However, long-term high expression of miR146a may severely attenuate the production of anti-viral cytokines as well as TNF-α, which would delay viral clearance and increase host susceptibility to chronic HBV infection. So, targeting HBV-induced miR-146a may facilitate anti-HBV response by reversing suppressed anti-HBV immunity.

To validate our hypothesis, we compared the expression of PPRs associated with virus recognition, and found that the RNA-sensor receptors RIG-I and MDA5, as well as the enhancer of the RIG-I signaling pathway RIG-G were down-regulated in HBV^+^ cells and patient tissues (Fig. 1), whereas TLRs did not show significant difference (data not shown). Meanwhile, HBV genome transfection could inhibit the transcription of IFN-β enhanced by RIG-I or RIG-G overexpression. Consistent with previous results, mature miR146a as well as its primary transcript and stem-loop precursor could be induced by HBV infection (Fig. 2), indicating that miR146a might be detrimental to RLR signaling. Indeed, miR146a mimics suppressed RIG-I expression and IFN-β production; conversely, miR146a inhibitor enhanced RIG-I expression and IFN-β production, as well as the responsiveness to RIG-I activator 3p-RNA (Fig. 3). Further investigation demonstrated RIG-I and RIG-G were post-transcriptional regulated by miR146a (Fig. 4), but miR146a did not bind to HBV itself according to bioinformatics analysis (Table S3). Encouragingly, blocking miR146a by an anti-sense or sponge strategy obviously inhibited HBV replication, transcription and capsid expression both in HBV-persistence mouse model and in HepG2.2.15 cells, accompanied with the increase of RIG-I and RIG-G expression in primary hepatocytes and restoring type I IFN production (Figs 5 and S4). By the way, miR146b may be not involved in HBV transcription and translation (Fig. S5).

Chronic HBV infection reflects a failure of the host’s immune system to control infection. One of the concerns in clinic for CHB treatment is to reverse immune tolerance status and trigger host immune defense against HBV. Since innate immunity is critical for protecting the host from HBV infection and persistence, innate immune pathways such as PRR signaling might be an optimum target for designing novel drugs against HBV infection. In 2009, experimental evidence showed ectopically expressed RIG-I CARD domain effectively promoted HBV clearance, suggesting that activation of RIG-I pathways was effective to inhibit HBV replication^3^. Interestingly, at the end of 2014, RIG-I was proved to possess dual functions during HBV infection, acting as an innate sensor and a direct anti-HBV factor^36^. Therefore, RIG-I-mediated intrinsic recognition of HBV and the following signal pathways might be a pivotal intracellular anti-HBV mechanism in hepatocyte and play a predominant role in host-HBV interaction. Based on these, we expect that eliminating inhibitory factor of RIG-I pathways would be a promising strategy for designing new anti-HBV immunopharmaceutical agents.

**Figure 6:**
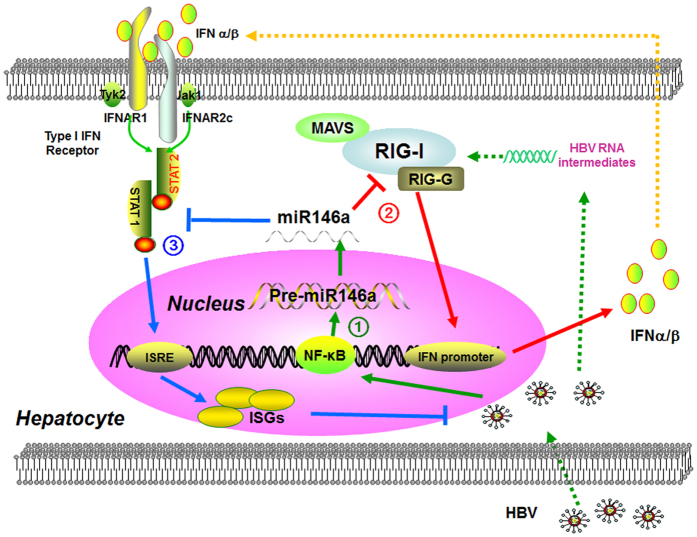
Working model. (1) HBV infection promoted endogenous miR146a transcription in hepatocytes. (2) miR146a directly targeted RIG-I and RIG-G mRNA, and then impaired RIG-I pathway axis-induced type I IFN production. (3) miR146a also inhibited STAT1 in a sequence-dependent manner, attenuates IFN-induced anti-viral genes expression and ultimately weaken anti-HBV response.

In this study, we showed that HBV-induced miR146a could posttranslationally inhibit both RIG-I and RIG-I enhancer (RIG-G) to negatively regulate cell-intrinsic innate immunity, leading to suppressing both type I IFN production and downstream ISG response (Fig. 6), resulting in impaired anti-viral innate immune responses against HBV. Moreover, antagonizing miR146a reversed immune tolerance and generated efficient anti-HBV immunity without immune injury. These evidences suggested miR146a might be a critical target for improving HBV clearance, providing a clue to develop novel preventive and therapeutic strategies for HBV infection in the future.

## Materials and Methods

### Cell lines and clinical samples

HepG2 and HepG2.2.15 HCC cell lines were both grown in complete DMEM (GIBCO, USA) supplemented with 10% fetal bovine serum (FBS). NKL (an NK-derived leukemia cell line that expresses high miR146a level^37^) and the HEK293 cell line were maintained in RPMI-1640 medium (GIBCO, USA) supplemented with 10% FBS. All cultures were incubated at 37 °C in a humidified atmosphere containing 5% CO_2_. Human liver samples from 7 HCC patients and 1 intrahepatic cholangiocarcinoma (ICC) patient were obtained from Qilu Hospital of Shandong University (the clinical profiles of study subjects are listed in Table S1).

### Ethics Statement

For the studies involving human participants, all experimental guidelines and protocols were approved by Ethical Committee of Shandong University. The methods were carried out in accordance with the approved guidelines. All written informed consents were obtained from all subjects in this study.

The animal study proposal and protocol were approved by Ethical Committee of Shandong University. Animal care provided was conformed to the principles expressed in “Guide for the Care and Use of Laboratory Animals” (NIH, 1996). All animal experimental protocols were performed in accordance with “Regulations for the Administration of Affairs Concerning Experimental Animals” (Approved by State Science and Technology Commission, People’s Republic of China, 10/31/1988).

### Plasmids

pAAV/HBV1.2 plasmid containing 1.2 copies of full-length HBV genome was kindly provided by Professor Pei-Jer Chen. pcDNA5-RIG-I CARD was kindly provided by Professor Ju-Tao Guo. pGL3-IFN-β-luc reporter vector was generously provided by Professor Zhijian Chen; LMP plasmid was kindly provided by Yuekang Xu. pIRESpuro3-RIG-G vector was generously provided by Dr. Kathryn C. Zoon. pGL3-basic and pRL-TK plasmids were purchased from Promega (Hongkong), and pMIR-REPORT™ Luciferase Assay System was purchased from Ambion (USA). pcDNA3 was purchased from Invitrogen (USA). pcDNA3-hsa-miR146a (Cat#15092) constructed by David Baltimore’s lab, were purchased from Addgene (USA). The plasmid LMP-HBV1.2 was constructed by inserting a segment including puromycin resistance gene and GFP gene digested by Sal I and Sph I from LMP vector into the vector pAAV/HBV1.2.

In order to inhibit endogenous miR146a contiguously, a miR146a sponge vector named pSIREN-PGK-miR146a-sponge was constructed based on pSIREN-Shuttle vector. “miR146a sponge” sequence “AACCCATGGAAAAGAGTTCTCA” was inserted as 10 tandem duplications. A cassette including puromycin resistance gene and GFP gene was also inserted into pSIREN-Shuttle at I-*Ceu* I site for tracing.

To construct 3′-UTR luciferase reporter plasmids of RIG-I and RIG-G, human RIG-I 3′-UTR fragment (770 bp), RIG-G 3′-UTR fragment (193 bp) containing the putative miR146a binding site were amplified from HepG2.2.15 cDNA, using clone primers as follows: RIG-I UTR, ATAACGCGTTGACCATTTTCCTATAATGATG (forward) and ACGAAGCTTGAGAGAGTGTGTGTAAAGGAG (reverse), and RIG-G UTR, ATAACGCGTTTACATAGTCCGAAGGTCTTACAAC (forward) and ACGAAGCTTGAATTCGAAGTCTGGATGAAAATG (reverse), cloned into downstream of luciferase gene. To introduce four base pair mutants in the seed region of miR146a binding site, we used an over-lap PCR approach while the primers were changed in the seed sequences. The plasmids were named RIG-I U (pmiR-reporter- RIG-I 3′-UTR WT), RIG-I U m1 (pmiR-reporter-RIG-I 3′-UTR site 1 mutant), RIG-I U m2 (pmiR-reporter-RIG-I 3′-UTR site 2 mutant), and RIG-I U m1+m2 (pmiR-reporter-RIG-I 3′-UTR site 1 and 2 mutants); RIG-G U (pmiR-reporter-RIG-G 3′-UTR) and RIG-G U m (pmiR-reporter-RIG-G 3′-UTR mutant). All the plasmids were confirmed by sequencing. Clone vector (pEasy-T series) and site mutant kit (Fast Mutagenesis System) were provided by TransGen Biotech (Beijing, China).

### Ribonuclease protection assay

miRNA probe sequences were designed based on published sequences listed in the miRBase Version 13.0 database. Non-isotopic RNA probes for the mature miR146a, U6 and control probes were synthesized using the mirVana^TM^ miRNA Probe Construction Kit (Ambion, USA) to incorporate DIG-labeled UTP (Cat#11209256910, Roche, Germany). The DNA templates for *in vitro* transcription are as follows: U6 Probe, ATATGGAACGCTTCACGAATTCCTGTCTC, miR146a, TGAGAACTGAATTCC ATGGGTTCCTGTCTC, and RPA were performed to evaluate the levels of mature miR146a using the mirVana^TM^ miRNA Detection Kit (Ambion, USA) according to the manufacturer’s instructions.

### *In vitro* transcription

The 3p-RNA ligand to trigger RIG-I signaling and the RNA probes for the RPA assay were both synthesized using the MEGAscript^®^ T7 kit (Ambion, USA). The DNA templates sequences were: 3p-RNA-Sense, GATCACTAATACGACTCACTATAGGG TAAGGCTATGAAGAGATAC (sense), 3p-RNA-Antisense, GATCACTAATACGAC TCACTATAGGGGTATCTCTTCATAGCCTTA (sense).

### siRNA, miRNA mimics and inhibitors

siRNA, miR146a/b mimics (dsRNA oligonucleotides with the same sequence of mature miR146a/b) and miR146a/b inhibitors (2′-OMe chemically modified single-stranded oligonucleotides with the reversal-complementary sequence of mature miR146a/b) were supplied by GenePharma (Shanghai, China). The siRNA sequences used for silencing RIG-I and RIG-G are listed as follows: RIG-I, AUCACGGAUUAG CGACAAA (sense), RIG-G-sense 1, GGCAAGCUGAAGAGUUAAU (sense), RIG-G-sense 2, GGGACUGAAUCCUCUGAAU (sense), Negative Control (NC), UUCUCCGAACGUGUCACGU (sense).

### Transfection

HepG2.2.15 or HepG2 cells were seeded into either 6- or 12-well plates, and then co-transfected with miR146a mimics/inhibitors and plasmids with LipofectamineTM 2000 (Invitrogen, USA) according to the manufacturer’s instructions. The miRNA mimics/inhibitors were introduced at final concentration of 20, 50 or 100 nM, and the transfection efficiency of mimics and inhibitors was shown in Figs S1,S2. The LMP-HBV1.2 plasmids were used at final concentration of 1 μg/mL. Negative controls for miRNA mimics (mNC), inhibitors (iNC) or siRNA (NC) were also transfected as experimental controls.

### Isolation and culture of primary mouse hepatocytes

Primary mouse hepatocytes were isolated and cultured as previously described^38^.

### RNA isolation and qRT-PCR analysis of mRNA and miRNA

RNA isolation and qRT-PCR analysis were performed as previously described^39^. To rule out any possible excess LMP-HBV1.2 plasmid contamination in HBV DNA and mRNA quantitation, 2 steps were performed during processing of all samples treated with the HBV-genome plasmid. In the first step, after plasmid transfection, the wells were washed 3 times with PBS before adding new medium to remove any residual untransfected plasmids or plasmids that only adhered to the cell membrane. We performed the second step before cDNA preparation, where total RNA was treated with DNase I at 37 °C for 30 min to digest any potential genomic DNA and plasmid. DNA-free RNA was then isolated again by phenol/chloroform extraction, re-precipitated and re-suspended, followed by the normal cDNA synthesis procedure. The miR146a precursor and primary transcript were reverse-transcribed by random N6 primers. All PCR primers^28^,^40^ are listed in Table S2 of the Supporting Information. SYBR Green Real-time PCR Master Mix (Code No. QPK-201) was provided by Toyobo, Japan.

### Luciferase reporter gene assay

To test IFN-β promoter activity, HepG2 or HepG2.2.15 cells were plated at a density of 1.5 × 10^4^ cells/well in 96-well plates and transiently co-transfected with 0.05 μg pGL3-IFN-β-luc reporter and a 0.02 μg pRL-TK plasmid expressing Renilla luciferase (to normalize transfection efficiency) accompanied by 0.05 μg pcDNA5-RIG CARD, pAAV/HBV1.2 or pIRESpuro3-RIG-G as well as 100 nM siRNA/miRNA using Lipofectamine^TM^ 2000.

For the miR146a target efficiency assay, HepG2, HepG2.2.15 and HEK293 cells were plated at a density of 1.5 × 10^4^ cells/well in 96-well plates and transiently co-transfected with 0.1 μg of the pmiR-Reporter empty vector or pmiR-Reporter working vectors (containing the WT or mutant nucleotides for the target region) plus miRNA mimics (50 nM) or miRNA inhibitors (100 nM), together with 0.02 μg of pRL-TK. After 36 h, the transfected cells were washed, lysed and evaluated for luciferase expression using the Dual-Glo Luciferase Assay System (Promega, USA).

### Preparation of the DIG (Digoxigenin)-labeled probe

The primer sequences for synthesizing the DIG-labeled HBV (HBVx gene), human β-actin and mouse GAPDH probes are as follows: HBVx, ATGGCTGCTAGGCT GTACTG (forward) and TTAGGCAGAGGTGAAAAAGTTGC (reverse), human β-actin, GTGGGGCGCCCCAGGCACCA (forward) and CTCCTTAATGTCACG CACGATTTC (reverse), mouse GAPDH, ACCATCTTCCAGGAGCGA (forward) and AGTGAGCTTCCCGTTCAGC (reverse). The DNA fragments corresponding to these genes were amplified using high-fidelity Taq PCR polymerase (Cat NO. E009, Novoprotein/ SinoBio, Shanghai, China) and then DIG-labeled with DIG-11-dUTP using the DIG-High Prime DNA Labeling and Detection Starter Kit II according to the manufacturer’s instructions (Roche Diagnostics GmbH, Germany).

### Southern Blotting

Total DNA from mouse liver tissues and HepG2.2.15 cells was extracted using the Universal Genome DNA Isolation Kit (Shengong Biotechnology Limited, Shanghai, China). DNA was then precipitated and dissolved in ddH_2_O. Total DNA (20 mg) was digested by HindIII and electrophoresed on a 1% agarose gel in 1 × TAE and transferred to Hybond^TM^-N + membranes (Amersham Bioscience, GE Healthcare, Buckinghamshire, UK). After crosslinking (HL-2000 HybriLinker, UVP, USA), the membrane was hybridized with denatured DIG-labeled HBx gene probe in DIG Easy Hyb buffer. The blot was incubated with specific probe for 20 hours at 42 °C^41^. The membranes were then blocked, incubated with diluted anti-DIG AP conjugate, washed, and visualized by CSPD (Roche Diagnostics GmbH, Germany) according to the protocol for the DIG High Prime DNA Labeling and Detection Starter Kit II (Roche Diagnostics GmbH, Germany). The images were examined by the ChemDoc™ XRS^+^ Imager using ImageLab^TM^ software, Version 3.0 (Bio-Rad Laboratories, USA).

### Northern Blotting

Total RNA from mouse liver tissues or HepG2.2.15 cells was isolated using Trizol (Invitrogen, USA) according to the manufacturer’s instructions. RNA samples were treated with DNase I (Ambion) for 30 min at 37 °C to clean up contaminated genomic DNA or plasmids, followed by phenol/chloroform extraction, re-precipitation and re-dilution. For Northern blot, 30 μg of RNA was electrophoresed on a 2% formaldehyde agarose gel in 1 × MOPS, transferred to a membrane, blotted with a DIG-labeled probe and visualized as described above. The membrane was also hybridized with a DIG-labeled mouse GAPDH or human β-actin probe to provide loading controls.

### Immunohistochemical staining

Immunohistochemical staining for HBsAg and HBcAg protein expression in mouse liver tissue was carried out as previously described^38^.

### Analysis of HBV DNA

Viral DNA levels in HCC cell supernatants and mouse serum were quantified by qRT-PCR according to the manufacturer’s instructions of the Diagnostic Kit for Quantification of HBV DNA (Da-An, Guangzhou, China). Mouse serum samples were first cleared by undergoing a free-nucleic acid clean-up procedure and then subjected to DNA extraction, as described previously^41^.

### ELISA

HBsAg and HBeAg levels in HCC cell supernatants and mouse serum were evaluated using ELISA kits from RongSheng Biotechnology (Shanghai, China) according the manufacturer’s instructions. For mouse liver IFN-α and IFN-β quantitation, 100 mg of mouse liver was homogenized in 0.5 ml tissue lysis buffer (20 mM Tris–HCl (pH 8.0), 5 mM EDTA, 3 mM EGTA, 1 mM DTT, 1% SDS, 1 mM PMSF) by Tissue Homogenizer (OSE-Y10, Tiangen, Beijing, P.R. China) at 4 °C. Samples were then centrifuged at 12000 rpm for 10 min, and the supernatants were collected for measurement using VeriKine^TM^ Mouse Interferon Alpha ELISA Kit (Product #42120) and Interferon Beta ELISA Kit (Product #42400) (PBL Biomedical Laboratories, Piscataway, USA). Human IFN-β in the supernatant of HepG2 cells was assayed using Human IFN-β ELISA Kit (Product #10-60-774, Y-Y Shanghai Chemical Reagent Co., Ltd, Shanghai, China).

### Western blot analysis

Rabbit monoclonal anti-RIG-I (Cat#3743) and anti-MDA5 (Cat#5321) were purchased from Cell Signaling Technology, Inc. (Danvers, MA, USA). Mouse monoclonal anti-human β-actin (SC-47778), anti-RIG-G (SC-133687) and anti-IFIT1 (SC-134948) were obtained from Santa Cruz Biotechnology (Santa Cruz, CA, USA). Protein bands were visualized by Immobilon Western Chemiluminescent HRP Substrate (Millipore, USA) and then captured and analyzed using ChemiDoc™ XRS^+^ Imager with ImageLab software, Version 3.0 (Bio-Rad Laboratories, USA).

### Statistical analysis

Statistical analysis was performed using the unpaired Student’s t-test. A value of *p* < 0.05 was considered statistically significant.

## Additional Information

**How to cite this article**: Hou, Z. *et al.* Hepatitis B virus inhibits intrinsic RIG-I and RIG-G immune signaling via inducing miR146a. *Sci. Rep.* **6**, 26150; doi: 10.1038/srep26150 (2016).

## Supplementary Material

Supplementary Information

## Figures and Tables

**Table 1:** Predicted matches between anti-viral factors and human miR146a.

Anti-viral factors	miR146a seed match			
	PicTar	TargetScan	RNAhybrid	miRanda
APOBEC3B (NM_004900)	None	None	None	None
APOBEC3F (NM_145298)	None	None	Yes	None
APOBEC3G (NM_021822)	None	None	None	None
MxA (NM_001144925)	None	None	3p	None
OAS1 (NM_016816)	None	None	None	None
OAS2 (NM_016817)	None	None	Yes, including 3p	None
OAS3 (NM_006187)	None	None	Yes, including 3p	Yes (BC012015)
RNase L (NM_021133)	None	Yes	Yes, including 3p	Yes
PKR (NM_002759)	None	None	None	None
RIG-I (NM_014314)	None	Yes	Yes	Yes
ISG15 (NM_005101)	None	None	None	None
ISG20 (NM_002201)	None	None	3p	None
IFIT1 (NM_001548)	None	None	Yes, including 3p	None
IFIT2 (NM_001547)	None	None	3p	None
RIG-G (NM_001031683)	None	Yes	Yes, including 3p	Yes
IFIT5 (NM_012420)	None	None	3p	Yes
TRIM22 (NM_006074)	None	None	Yes	None
TLR3 (NM_003265)	None	None	Yes	None
TLR7 (NM_016562)	None	None	3p	None

Nineteen immune genes, including innate immune receptors, host restriction factors and ISGs, were submitted to bioinformatic analysis for potential interaction with the miR146a family using PicTar, TargetScan, RNAhybrid and miRanda.
