# Supplementary material for: Hepatitis B virus inhibits intrinsic RIG-I and RIG-G immune signaling via inducing miR146a
Source: Sci Rep. 2016 May 23;6:26150. doi: 10.1038/srep26150 (PMC4876503; doi:10.1038/srep26150)
Supplement: Supplementary Information [file srep26150-s1.pdf]

Supplementary Information

Hepatitis B virus inhibits intrinsic RIG-I and RIG-G immune signaling via inducing miR146a

Zhaohua Hou, Jian Zhang, Qiuju Han, Chenhe Su, Jing Qu, Dongqing Xu, Cai Zhang, Zhigang Tian

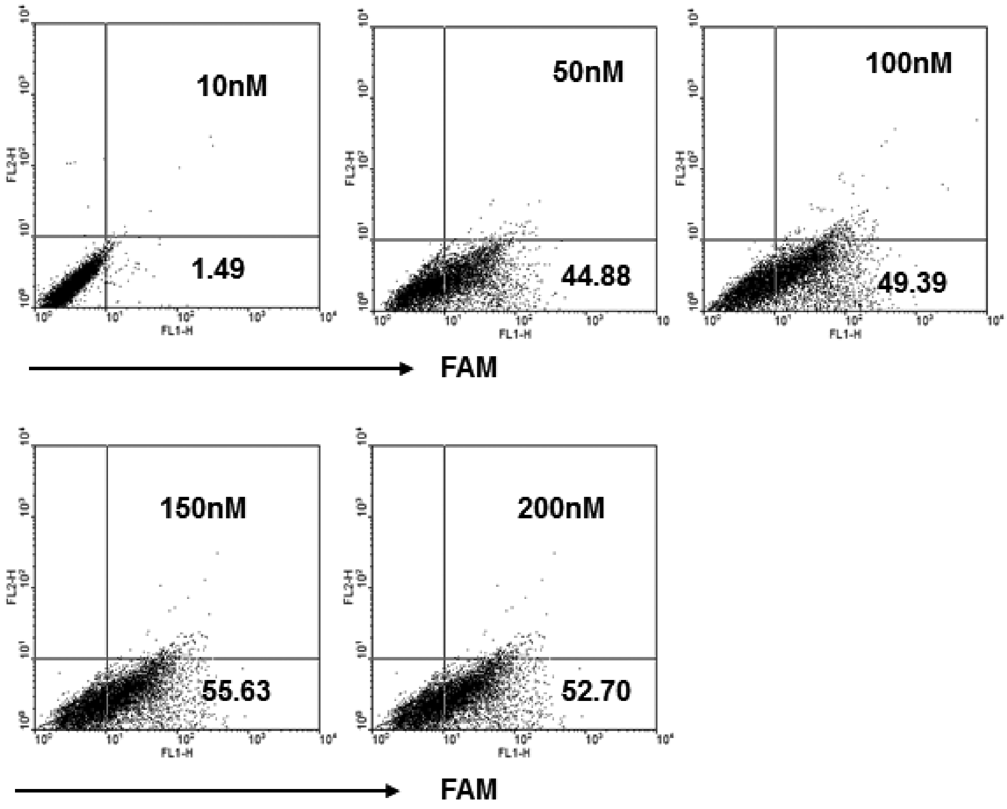

Fig. S1 Transfection efficiency of FAM-labeled miRNA mimics at the concentrations of 10nM，50nM，100nM，150nM and 200nM by Lipofectamine 2000 in HepG2 cells.

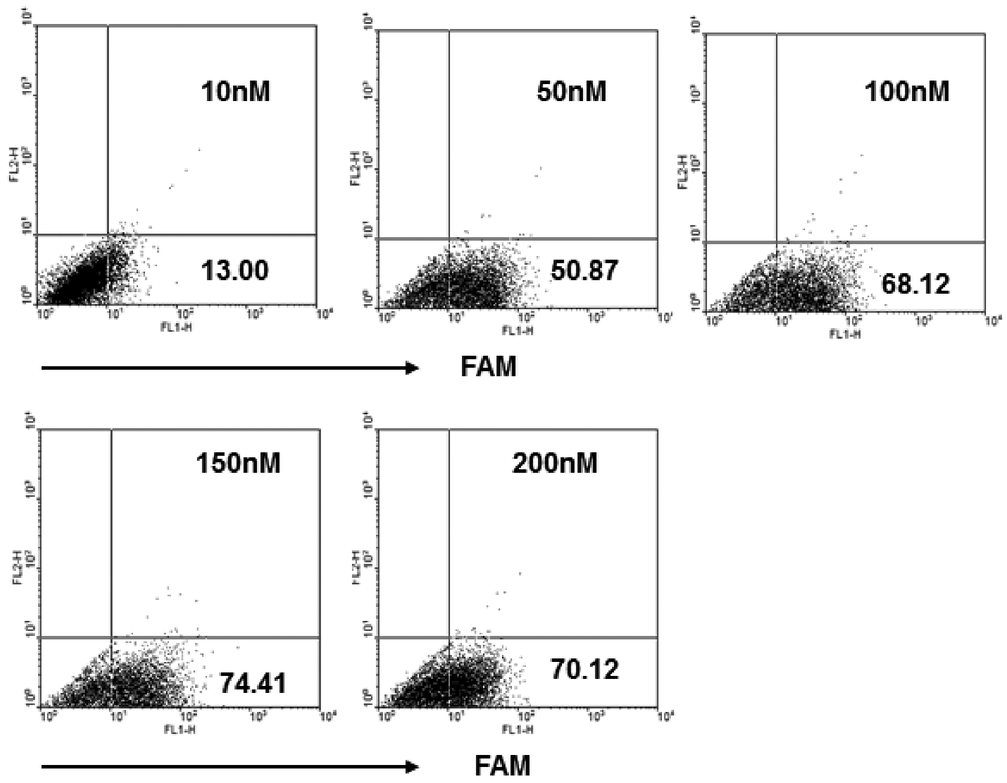

Fig. S2 Transfection efficiency of FAM-labeled miRNA inhibitors at the concentration of 10nM，50nM，100nM，150nM，200nM by Lipofectamine 2000 in HepG2.2.15 cells.

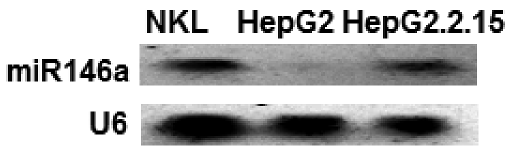

Fig. S3. Mature miR146a amount in HepG2 and HepG2.2.15 cells. miR146a was directly measured by an RPA assay. The NKL cell line was used as a positive control, and U6 was used as the loading control.

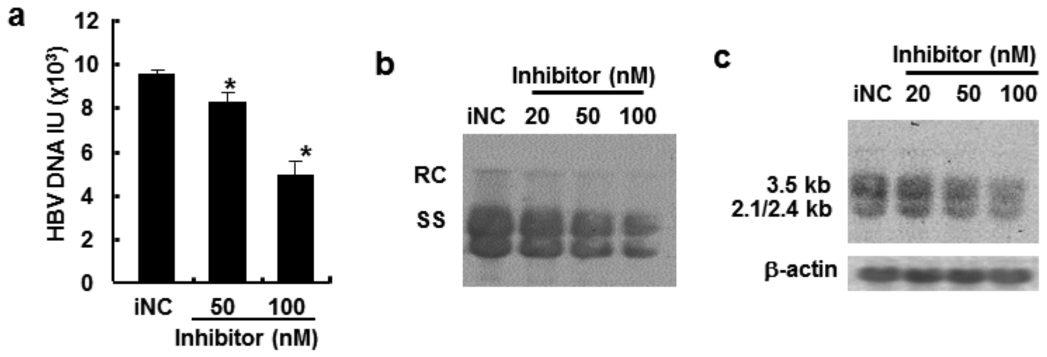

Fig. S4. miR146a reduced HBV load *in vitro*. HepG2.2.15 cells were transfected with miR146a mimics/inhibitors at doses ranging from 20–100 nM. (a) After 48 hours, HBV DNA load was determined by qRT-PCR. Data are expressed as the mean ± SD from at least 3 independent experiments. $*p < 0.05$: versus control vector or the RNA-transfected negative control group. (b, c) Total HBV DNA and RNA levels were analyzed by (b) Southern blotting and (c) Northern blotting with the same HBV probe respectively. Human β-actin was used as a loading control for the Northern blot experiment.

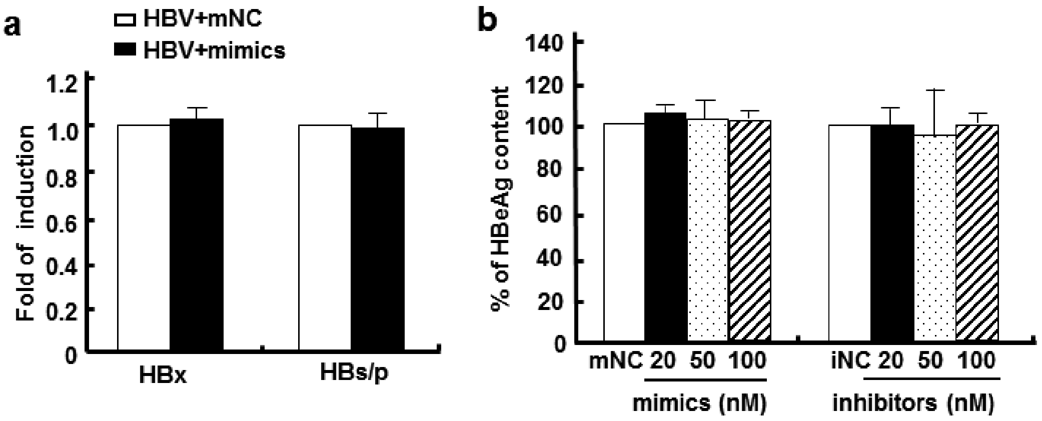

Fig. S5. miR146b had no impact on HBV transcription and translation. (a) HepG2 cells were co-transfected with pAAV-HBV1.2 plasmid (1 μg/mL) and 20 nM miR146b mimics or negative control (mNC). Then, HBx and HBs/p mRNA levels were measured by qRT-PCR 48 hours later. (b) HepG2.2.15 cells were transfected with miR-146b mimics/inhibitors at doses ranging from 20–100 nM, and HBeAg levels were measured by ELISA 48 hours after treatment. Data are expressed as the mean ± SD from at least 3 independent experiments. *$p < 0.05$: versus control-RNA-transfected group.

Table S1 Clinical profiles of study subjects

	**No.1**	**No.2**	**No.3**	**No.4**	**No.5**	**No.6**	**No.7**	**No.8**
Age	49	68	65	62	57	45	58	65
Gender	Male	Male	Male	Male	Male	Male	Male	Male
HBV Infection	+	+	+	+	-	-	-	-
ALT	34	27	60	52	48	38	45	26

No. 1-4, HBV^{+} HCC patients; No. 5-7, HBV^{-} HCC patients; No. 8, ICC patient.

Table S2 Sequences of primers used for real-time PCR analysis

Target sequence	Sequence 5′–> 3′	size (bp)
pre-hsa-miR146a[40]	F: CCGATGTGTATCCTCAGCTTTG<br>R: GCTGAAGAACTGAATTTCAGAGGTC	79
pri-hsa-miR146a[28]	F: TGAGAACTGAATTCCATGGGTT<br>R: ATCTACTCTCTCCAGGTCCTCA	106
hIFN-α[6]	F: CTCCTTTCTCCTGCCTGAAG<br>R: AAGTGTCTCATCCCAAGTAGC	170
hIFN-β[6]	F: TGCTCTCCTGTTGTGCTTCTCC<br>R: CATCTCATAGATGGTCAATGCGG	222
hTNF-α[6]	F: ATCTTCTCGAACCCCGAGTGA<br>R: GAGGGCTGATTAGAGAGAGGTC	83
hU6	F: CTCGCTTCGGCAGCACATA<br>R: AACGCTTCACGAATTTGCG	94
HBV x[6]	F:CCGTCTGTGCCTTCTCATCTGC<br>R: ACCAATTTATGCCTACAGCCTCC	256
HBV s/p[6]	F:ATCCTGCTGCTATGCCTCATCTT<br>R: ACAGTGGGGGAAAGCCCTACGAA	314
mRIG-I	F: CCACCTACATCCTCAGCTACATGA<br>R: TGGGCCCTTGTTGTTCTTCT	86
mRIG-G	F: CCTACATAAAGCACCTAGATGGC<br>R: ATGTGATAGTAGATCCAGGCGT	149
hGAPDH[6]	F: GAAGGTGAAGGTCGGAGT<br>R: CATGGGTGGAATCATATTGGAA	155

Table S3 Bioinformatic analysis of interaction between miR146a and HBV genome

HBV Genotype	Predicted seed match	
	miR146a-5p	miR146a-3p
adr(M38636.1)	None	None
ayr(NC003977.1)	None	None
ayw (M57663.2)	None	None
ayw (U95551.1)	None	None
